# Influence of Jojoba seed waste and carbon black hybrid filler on styrene butadiene rubber composites characteristics

**DOI:** 10.1038/s41598-025-21649-4

**Published:** 2025-10-17

**Authors:** T. A. Zidan, A. I. Khalaf, A. A. Ward

**Affiliations:** 1 Polymers and Pigments Department, National Research Centre, 33 El-Buhooth Street, Dokki, 12622 Giza Egypt; 2 Microwave Physics and Dielectrics Department, National Research Centre, 33 El-Buhooth Street, Dokki, 12622 Giza Egypt

**Keywords:** Hybrid filler, Jojoba seed, Carbon black, Styrene-butadiene rubber, Viscoelastic and dielectric properties, Chemistry, Materials science, Physics

## Abstract

This study investigates the effects of jojoba seed waste /carbon black hybrid filler on the mechanical, dielectric, and viscoelastic properties of styrene butadiene rubber composites. The ratios of the seed to carbon black are 0/50, 10/40, 20/30, 30/20, 40/10, and 50/0 phr, respectively. The study evaluates rheometric characteristics and mechanical properties, specifically tensile strength, elongation, and modulus at 100% elongation, both before and after thermo-oxidative aging at 90 °C for various periods of two, four, and six days. Further assessments involved hardness, swelling behavior, crosslink density, and water uptake as well. Moreover, Fourier transform infrared spectroscopy was used to characterize the chemical composition of the samples, while field emission scanning electron microscopy was used to evaluate the surface morphology. The results demonstrate that, increasing the ratio of jojoba seed leads to decreased viscosity and scorch time, improved elongation, and reduced tensile strength, modulus, and hardness. Filler agglomeration alters crosslinking, mechanical characteristics, and aging resistance. Measurements of water uptake demonstrated a positive correlation with seed content, ascribed to the hydrophilic characteristics of the hybrid filler. The dielectric study indicated that increased seed concentration led to a reduction in dielectric permittivity and electrical conductivity, implying enhanced insulating properties. Dynamic mechanical analysis revealed decreases in storage modulus E′ and damping factor tan δ, indicative of enhanced molecular mobility and a reduced glass transition temperature Tg, consistent with increased to the plasticizing influence of residual jojoba oil. In addition, viscosity-related parameters exhibited similar trends, signifying increased flexibility throughout the temperature spectrum. These findings highlight the trade-offs between flexibility and stiffness, allowing composite qualities to be tuned for a wide range of applications.

**Supplementary Information:**

The online version contains supplementary material available at 10.1038/s41598-025-21649-4.

## Introduction

The practice of using fillers to reinforce rubber is very significant from a practical and technological standpoint^1^. Rubber compositions require the addition of fillers, or reinforcements, such as carbon black, clays, and silicates, to encounter the requirements for certain material qualities like tensile strength and abrasion resistance. Thus, in terms of formulation, selecting the right filler is just as crucial as selecting rubber in order for a product to meet its performance requirements^2^. Nonetheless, the rubber industry continues to face difficulties due to the ongoing need for novel, inexpensive, lightweight, and environmentally friendly reinforcing filler^1^.

The most basic hybrid filler is carbon–silica filler (CSF). Silica and carbon black are doped with one another, which enhances their dispersion in the rubber matrix and successfully lowers the filler-filler contact. Wang et al.^3–5^ published a number of several studies on CSF-filled rubber; however, they did not thoroughly examine the static mechanical properties, filler-rubber interaction, or rubber network structure^6^, instead concentrating on the dynamic mechanical properties.

In reinforcement applications, natural fibers are starting to make sense as alternatives to synthetic fibers^7,8^. Synthetic fibers are expensive, non-biodegradable, and have limited recycling potential. Natural fibers are gaining ground on synthetic fibers despite having a lower strength^9,10^. Their affordability, light weight, biodegradability, high modulus, availability of specific strength, and gas emissions are the reasons for this. Natural fiber production releases little to no carbon dioxide in comparison to synthetic fiber processing^11^. Because naturally occurring cellulosic fibers are naturally eco-friendly, they are an excellent choice for reinforcement for creating bio-composite materials^12,13^.

A natural fiber is powdered jojoba seed waste. For rubber products, it serves as an environmentally beneficial antioxidant^14^. The authors discovered that adding the powdered jojoba seed to silica-NBR composites improved the curing, mechanical, and swelling properties of acrylonitrile butadiene rubber, compared to the composite made with the commercial antioxidant poly(2,2,4-trimethyl-1,2-dihydroquinoline) (TMQ). They also discovered that the ideal concentration of jojoba powder was 4 phr. Soliman et al.^15^ investigated the antibacterial activity and dielectric characteristics of jojoba seed waste powder (JSWP) in nitrile butadiene rubber. It has been discovered that jojoba seed waste exhibit strong antibacterial properties in rubber. The addition of JSWP increased the resultant composites’ permittivity, dielectric loss, and conductivity, according to the dielectric results. They found that JSWP is important for NBR/JSWP compounds to have outstanding electrical performance. Furthermore, the produced rubber vulcanizates showed effective antibacterial activity against test strains of bacteria and fungi. On the other hand, the dielectric characteristics are the ability of a substance to store and disperse electrical energy when exposed to an electric field. These properties are defined by parameters such as permittivity, dielectric constant, and loss factor, which are affected by frequency, temperature, and moisture content. Dielectric materials are extensively explored for their use in electronics^16^, energy storage^17,18^, and sensing technologies^19,20^.

However, researchers frequently examine the dielectric properties of seed powder to evaluate its moisture content and overall quality. These properties are affected by the seed’ composition, particularly their oil and moisture levels. Research indicates that the dielectric constant of seed powder changes with variations in frequency and moisture content, making it a valuable factor for applications in agriculture and industry^21^. Jojoba seed, recognized for their high oil content, have distinctive dielectric characteristics. According to research, integrating jojoba seed meal powder into materials such as rubber composites improves their dielectric performance. The inclusion of jojoba seed powder increases permittivity, dielectric loss, and conductivity, making it useful for applications that require enhanced electrical features^15^. The aim of the present work is to study the effect of jojoba seed waste powder with carbon black as a hybrid filler for styrene butadiene rubber (SBR) composites on the rheometric characteristics, mechanical (before and after thermo-oxidative aging at 90 °C for different time periods), hardness, dielectric and dynamic mechanical properties of the composites. Moreover, swelling properties, crosslink density and water uptake are also discussed.

## Experimental

### Materials

Transport and Engineering Company (TRENCO) in Alexandria provided the styrene butadiene rubber (SBR 1502 non-staining) with a styrene content of 23.5%, specific gravity of 0.945, Tg with − 60 ± 1 °C, and Mooney viscosity M_L_ (1 + 4) of 52 ± 3 at 100 °C. As activators, 5.55–5.61 specific gravity zinc oxide and 0.90–0.97 specific gravity stearic acid were employed at 15 ± 1 °C. A commercial antioxidant called poly(2,2,4-trimethyl-1,2-dihydroquinoline) [TMQ] was employed. As a reinforcing agent, carbon black (high abrasion furnace; HAF) was utilized. Plasticizer utilized in the process was processing oil. An accelerator was employed: N-cyclohexyl-2-benzothiazole sulphenamide (CBS). It is a light gray powder with a melting point of 95–100 °C and a specific gravity of 1.27–1.31 at room temperature. Elemental sulfur was employed as vulcanizing agent. At room temperature, its specific gravity is 2.04–2.06, making it a fine, pale yellow powder. Jojoba seed were obtained from the Al– Kanz oil extraction company, Zagazig, Egypt. They were pressed in the oil unit at National Research Centre, Egypt and jojoba seed waste were obtained. It consists of many materials. Protein represents 22.9%, 1.2% crude oil, 15.4% crude fiber, 4.1% ash and 56.4% nitrogen free extract (NFE), on a dry weight basis. Also, there are some elements such as Ca (69.91 ± 1.1%), K (1312.60 ± 20.3%), Na (29.12 ± 1.07%), Mg (260.16 ± 1.19), Mn (4.1 ± 0.76%), Cu (1.79 ± 0.31%), P (350 ± 11.20%), Fe (8.86 ± 1.02%), and Zn (2.57 ± 0.87%)^22^. JSW was pulverized in a laboratory grinder and then sieved through a 230 μm screen to create the JSW powder, which is a fine powder that is easily combined with rubber materials.

### Instruments

#### Preparation of styrene butadiene rubber composites filled with Jojoba seed/carbon black hybrid filler

A two-roll mill with a 470 mm diameter was used to combine the SBR compositions that were designed using various components (Table 1). There was a 300 mm operating distance. The slow roller has a 24 r.p.m. speed and a 1:1.4 gear ratio. The rubber mixtures were left overnight to vulcanize. Utilizing a hydraulic hot press with a pressure of roughly 4 MPs. The rubber mixes are prepared according to the formulations represented in Table 1.

**Table 1:** Formulations of the SBR mixes filled with Jojoba seed waste powder/carbon black hybrid filler.

Ingredients (phr*)	S0/C50	S10/C40	S20/C30	S30/C20	S40/C10	S50/C0
SBR	100	100	100	100	100	100
ZnO	5	5	5	5	5	5
Stearic acid	2	2	2	2	2	2
Jojoba seed	0	10	20	30	40	50
Carbon black	50	40	30	20	10	0
Processing oil	5	5	5	5	5	5
TMQ	1.5	1.5	1.5	1.5	1.5	1.5
CBS	1.5	1.5	1.5	1.5	1.5	1.5
Sulfur	1.5	1.5	1.5	1.5	1.5	1.5

*phr: part per hundred parts of rubber.

#### Rheometric characteristics of styrene butadiene rubber composites filled with Jojoba seed waste/carbon black hybrid filler

The vulcanization process was carried out, and the molds were made in accordance with the unique cure times (TC_90_) ascertained by use of TA Instruments’ MDR one (Moving Die Rheometer); USA at 152 ± 1 °C for 30 min. The curing characteristics obtained from the rheometric graph were minimum torque (M_L_), maximum torque (M_H_), torque extent (ΔM = M_H_-M_L_), scorch time (TS_2_), and optimum cure time (TC_90_). From determining the values of TC_90_ and TS_2_, the cure rate index (CRI) was calculated as follows:

1 $${\text{CRI}}\,=\,{\text{1}}00/{\text{T}}{{\text{C}}_{{\text{9}}0}} - {\text{T}}{{\text{S}}_{\text{2}}}$$

#### Mechanical properties of styrene butadiene rubber composites filled with Jojoba seed waste/carbon black hybrid filler

Mechanical characteristics of the prepared SBR vulcanizates were assessed using an electronic Zwick tensile testing machine (model Z010) from Germany in accordance with ASTM D412. To measure the mechanical properties in terms of tensile strength (MPa), elongation at break (%) and modulus at 100% elongation (MPa), five samples are used and averaged. The shore hardness of the prepared composites was measured according to ASTM D2240 standard using a Bareiss Shore A durometer (Germany). All measurements were performed at room temperature 25 ± 1 °C.

#### Thermo-oxidative aging of styrene butadiene rubber composites filled with Jojoba seed waste/carbon black hybrid filler

Thermo-oxidative aging procedure was carried out on samples from SBR vulcanizates by placing them in an air-circulating oven set at 90 ± 1 °C for two, four, and six days, in accordance with ASTM D 572-04. The specimens were kept at 25 °C for 24 h before tensile tests were performed. Mechanical characteristics were measured using five samples and then the average is taken. The aging resistance is expressed by determining the retention percentage (%) in the tensile properties. Retention (%) is calculated by the following equation:

2 $${\text{Retention}}\left( {\%} \right)=\frac{{{\text{Mechanical property after aging}}}}{{{\text{Mechanical property before aging}}}}$$

#### Swelling and crosslink density

In accordance with ASTM D 573–2007, the prepared SBR composites were allowed to swell for a full day in toluene. Three samples are used and averaged. The following relation was used to compute the molecular weight between crosslinks (Mc) using the Flory-Rehner relation^23–30^.

3 $$\frac{1}{{\left( {2{\text{Mc}}} \right)}}=\frac{{ - 1}}{{{2_\rho }{V_R}}}{\text{x}}\left[ {\frac{{\ln \left( {1 - {V_R}} \right)+{V_R}+\mu {V_{{R^2}}}}}{{\left( {{V_{{R^{1/3 - 1/2}}}}{V_R}} \right)}}} \right]$$

where, the density of SBR (ρ) is 0.945 g/cm³, the interaction parameter between SBR and toluene (µ) is 0.45, and V_R_ represents the volume fraction of rubber in the swollen material; V_R_ is calculated according to the following formula: V_R_ = 1/(1 + Qm), where Qm = Q (equilibrium swelling percent)/100. The crosslink density ν is calculated using Eq. (4):

4 $$\nu \,=\,{\text{1}}/\left( {{\text{2Mc}}} \right)$$

#### Water uptake

The water uptake study was carried out in compliance with ASTM D570-95. To measure the water uptake, three samples are used and the average was taken. For different time periods, the vulcanized samples were immersed in distilled water. An electronic balance with an accuracy level of 0.5 mg was used to weigh each specimen at regular intervals. Prior to weighing, the samples were dried using tissue paper. The sample’s water content (Wc) was expressed as a weight%^31^. The following equation was used to calculate the water uptake:

5 $$Water~Uptake~\left( {Wc} \right),\% =\frac{{{\text{Wt}} - {\text{W}}0}}{{{\text{W}}0}}~~~~~ \times ~~~~~~100~~$$

where W_t_ is the weight of the specimen at time (t) and W_O_ is the initial weight of the sample before placing in water.

#### Fourier transform infrared spectroscopy

The instrument used to record the FTIR was a Jascow FTIR-430 from Japan. Attenuated Total Reflection (ATR) method was used to measure FTIR of the prepared composites. The spectrum range of 4000 to 400 cm^− 1^ was used to measure the data.

#### Field emission scanning electron microscope

Surface and cross-section images of the prepared SBR composites were taken by SEM, Quanta FEG-250.

#### Broadband dielectric spectroscopy

The Schlumberger Impedance / Gain-Phase Analyzer 1260, operating within the frequency range of 0.1 Hz to 1 MHz, was utilized to measure the permittivity ε′, dielectric loss ε″, and alternating resistance Rac at room temperature (30 ± 1 °C). A total of three samples are used for each measurement, and the average is then calculated. The error in ε′ and tan δ amounts to ± 1% and ± 3%, respectively. The temperature of the samples was controlled by a temperature regulator with (Pt 100) sensor. The error in temperature measurements amounts ± 0.5 °C.

#### Dynamic mechanical analysis

The glass transition temperature (Tg) of the samples under investigation was evaluated using a dynamic mechanical analyzer (DMA1) from Mettler Toledo, Switzerland. A tension mode of deformation was implemented. The samples were subjected to a cyclic tensile strain with a force amplitude of 0.1 N at a frequency of 1 Hz according to ASTM D4065. The storage modulus ((E′)), mechanical damping factor/loss factor (tan δ), and complex viscosity (η*) were measured within the temperature range of -80 to 120 °C at a rate of 5 °C per minute.

## Results and discussion

### Rheometric characteristics

Rheometric characteristics of the SBR composites filled jojoba seed waste and carbon black hybrid filler are shown in Table 2.

**Table 2:** Rheometric characteristics of the prepared SBR composites.

Sample	S0/C50	S10/C40	S20/C30	S30/C20	S40/C10	S50/C0
M_L_	1.62	1.35	1.08	1.11	0.99	0.41
M_H_	6.63	6.73	8.03	9.35	8.84	3.83
M_H_-M_L_	4.74	5.38	6.95	8.24	7.85	3.42
TS_2_	6.52	6.55	6.26	5.34	4.88	4.08
TC_90_	9.15	10.4	11.4	14.2	13.2	11.4
CRI	37.7	25.9	19.5	11.3	12.1	13.7

M_L_: Minimum torque; M_H_: Maximum torque; M_H_-M_L_: Torque extent; TS_2_: Scorch time; TC_90_: Optimum cure time; CRI: Cure rate index.

As illustrated in Table 2, M_L_ decreases gradually by increasing the ratio of seed in the hybrid filler, and this may be attributed to the compositional characteristics of jojoba seed waste that decreases the viscosity of compounded rubber and consequently increases the elasticity of vulcanizates^32^. M_H_ and M_H_-M_L_ show an increase by addition of 10 phr to 40 phr of seed and decrease again by addition of 50 phr of them. The increase in torque after rising seed concentration may be ascribed to increasing the interaction of the hybrid filler with the rubber matrix, along with enhanced filler distribution^14^. The decrease in M_H_ by addition of 50 phr of seed may be attributed to the presence of a percentage of oil that has a plasticizing effect on rubber mix, which leads to a reduction in crosslinking density^33^. TS_2_ is the scorch time, which measures the time at which the vulcanization of the samples starts. Also the scorch time decreases by increasing the percentage of seed. This reduction can be attributed to the increased crosslinking density within the rubber composites, which accelerates the onset of vulcanization^34^. TC₉₀ denotes the duration required for the rubber compound to get 90% of its complete cure, illustrating the kinetics of the vulcanization process^24–28^. Clearly, TC_90_ increased from 9.15 min (S0/C50) to a maximum of 14.2 min at S30/C20, then decreased to 11.4 min at S50/C0. This non-monotonic trend suggests delayed curing around the intermediate composition, followed by shorter cure durations at higher seed ratios. On the other hand, the Cure rate index (CRI) decreased significantly from 37.7 min⁻¹ (S0/C50) to 11.3 min⁻¹ (S30/C20) before plateauing, suggesting that the vulcanization reaction was retarded.

### Mechanical properties of styrene butadiene rubber composites filled Jojoba seed waste/carbon black hybrid filler

**Fig. 1:**
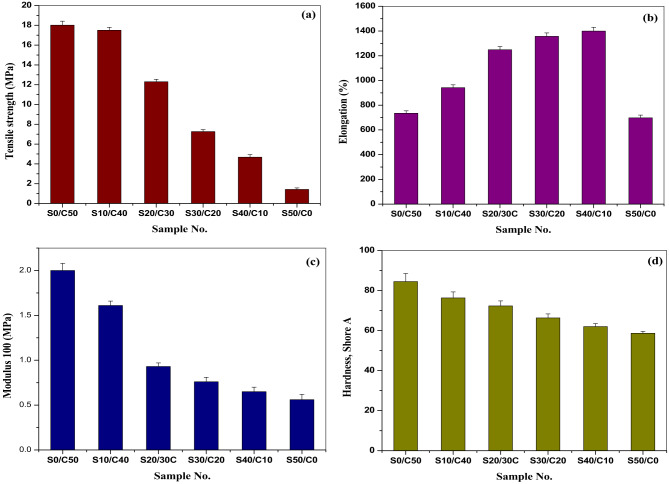
**(a-d)** Mechanical properties of SBR loaded with various concentrations of jojoba seed waste/carbon black hybrid filler; (a) Tensile strength, (b) Elongation, (c) Modulus at 100% elongation, and (d) Hardness, Shore A.

The physico-mechanical properties of SBR loaded with various concentrations of jojoba seed waste powder and carbon black (S0/C50; S10/C40; S20/30 C; S30/C20; S40/C10; S50/C0) are shown in Fig. 1(a-d). As illustrated in Fig. 1a, the tensile strength of the vulcanizates containing carbon black shows the highest value and this is due to the reinforcement effect of carbon black filler. By replacing 10 phr of carbon black with the seed, the tensile strength shows a slight decrease. Increasing, the concentration of the seed up to 50 phr, leads to a gradual decrease in the tensile strength. The decrease in tensile strength of the vulcanizates may be attributed to the presence of natural oil in jojoba seed waste that acts as a natural plasticizer^31^. The elongation at break (Fig. 1b) of the prepared vulcanizates increases by increasing the seed ratio in the seed/carbon black hybrid filler up to 40 phr of seed. This may be attributed to the plasticizing effect of jojoba seed waste due to the percentage of jojoba oil present in the seed^33^. The modulus at 100% elongation is shown in Fig. 1.(c). The modulus decreases gradually by increasing the concentration of seed in the hybrid filler. This is may be ascribed to the plasticizing effect of jojoba oil^33^.

Hardness of the composites is measured. The results are shown in Fig. 1(d). As observed, the values of hardness slightly decrease by increasing seed concentration in the hybrid filler.

### Thermo-oxidative aging of styrene butadiene rubber composites filled seed/carbon black hybrid filler

**Fig. 2:**
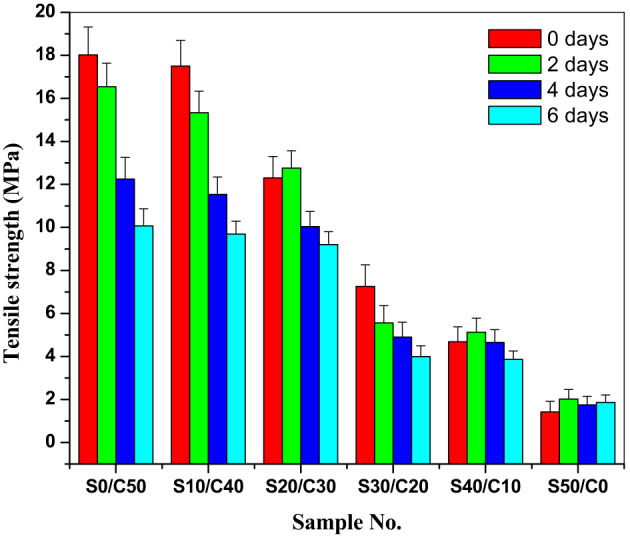
Tensile strength after aging of SBR loaded with various concentrations of jojoba seed waste/carbon black hybrid filler.

**Fig. 3:**
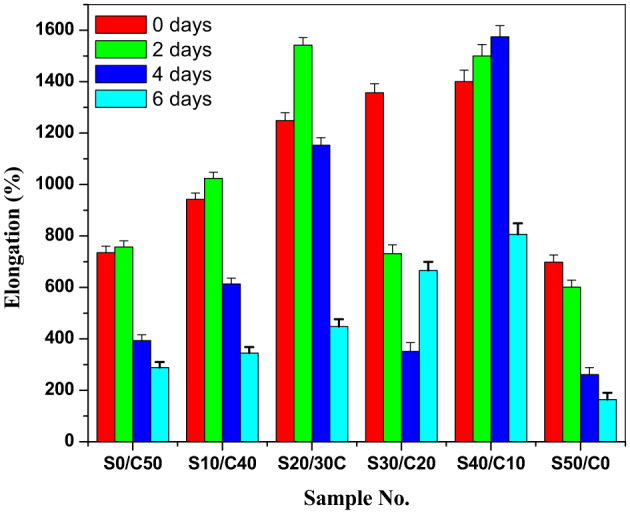
Elongation (%) after aging of SBR loaded with various concentrations of jojoba seed waste/carbon black hybrid filler.

**Fig. 4:**
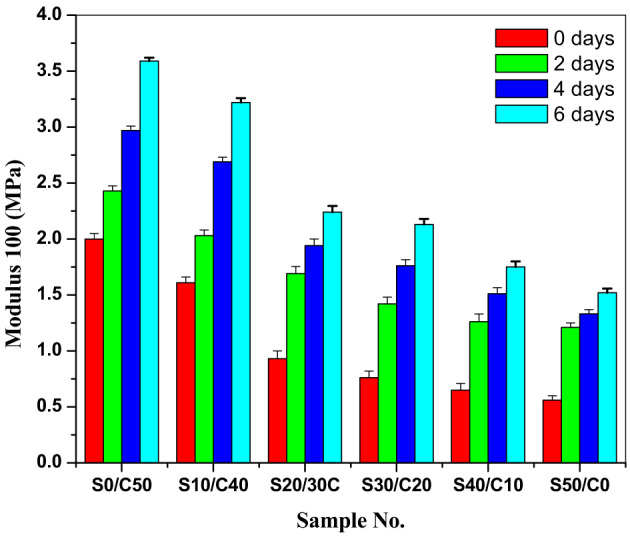
Modulus at 100% elongation after aging of SBR loaded with various concentrations of jojoba seed waste/carbon black hybrid filler.

The prepared SBR composites are subjected to thermo-oxidative aging at 90 °C for two, four, and six days. The values of tensile strength, elongation and modulus at 100% elongation are depicted in Figs. 2, 3 and 4, respectively and the retained values are illustrated in Table 3. As observed from Fig. 2, the tensile strength decreases by increasing aging time from two to six days. The resistance of the vulcanizates to aging is in the order of S0/C50˃ S10/C40˃S20/C30˃S30/C20˃S40/C10˃S50/C0.

The elongation at break is represented in Fig. 3. It shows appropriate results. The effect of aging on the elongation of SBR composites is in the order of S40/C10˃S30/C20˃S20/C30˃S10/C40˃S0/C50˃S50/C0.

The effect of aging on modulus at 100% elongation is illustrated in Fig. 4. As observed, the modulus increases by increasing aging time^24–28,35^. By aging, the polysulfide crosslinks within the rubber composites begin to deteriorate and more additional mono- and disulfide crosslinks begins to form within the composite. This process is known as the post-curing effect which results in an increase in the stiffness of the resulting SBR composites^14^. The effect of aging on modulus is in the order of S0/C50˃ S10/C40˃S20/C30˃S30/C20˃S40/C10˃S50/C0.

The retained values of the physico-mechanical properties (tensile strength, elongation and modulus) of the prepared SBR composites containing various concentrations of jojoba seed waste/carbon black hybrid filler are calculated according to Eq. (5) and represented in Table 3. As it is observed from the table, the composites show thermal stability by aging till six days.

**Table 3:** Retained values of mechanical properties of styrene butadiene rubber composites.

Sample code	S0/C50	S10/C40	S20/C30	S30/C20	S40/C10	S50/C0
Time, days	Retained of tensile Strength					
0	100	100	100	100	100	100
2	112	97	103	91	87	121
4	107	89	95	88	74	116
6	98	78	86	70	63	108

	Retained of elongation at break					
Time, days	S0/C50	S10/C40	S20/C30	S30/C20	S40/C10	S50/C0
0	100	100	100	100	100	100
2	107	104	120	117	102	112
4	87	81	106	100	77	94
6	45	42	59	55	40	51

	Retained of modulus at 100 elongation					
Time, days	S0/C50	S10/C40	S20/C30	S30/C20	S40/C10	S50/C0
0	100	100	100	100	100	100
2	151	174	187	200	218	125
4	185	208	232	243	264	158
6	220	240	280	288	297	199

The retention percentage of tensile strength values for the SBR composites decreases with increasing aging time periods. This may be attributed to the nature of jojoba seed^36^. The retained values of the physico-mechanical properties of SBR composites are improved after 6 days aging at 90 °C. All the properties are excellent and improved.

### Swelling properties and crosslink density of styrene butadiene rubber composites filled Jojoba seed waste powder /carbon black hybrid filler

**Fig. 5:**
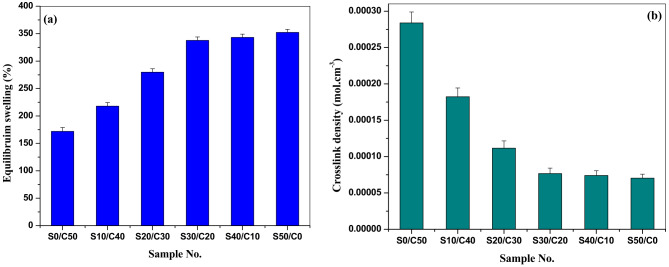
**(a**,** b)** (a) Equilibrium swelling, and (b) crosslink density of SBR loaded with various concentrations of jojoba seed waste/carbon black hybrid filler.

The SBR composites are swelled in toluene for 24 h. The equilibrium swelling is calculated and shown in Fig. 5(a). Increasing the concentration of seed in the hybrid filler leads to increasing the value of equilibrium swelling. This may be ascribed to a decrease in crosslinking of the vulcanized rubber by increasing the concentration of seed with the formation of long crosslinks that leads to penetration of increased amounts of toluene^26,28^.

Crosslink density is shown in Fig. 5(b). The figure shows the opposite behavior of equilibrium swelling. The crosslink density decreases gradually by increasing the concentration of seed in the hybrid filler. This may be due to decreasing the number of crosslinks formed during curing, leading to a decrease in crosslink density.

Descriptive statistical parameters, including mean values, standard deviations (SD), and the corresponding minimum and maximum ranges, were systematically computed for all graphical datasets (Figs. 1, 2, 3, 4 and 5), tabulated results (Tables S1–S9), and data presented in the supplementary section. These metrics serve to quantitatively capture the variability inherent in the measurements and substantiate the reproducibility of observed experimental trends^37,38^.

### Water uptake of styrene butadiene rubber composites containing Jojoba seed waste/carbon black hybrid filler

**Fig. 6:**
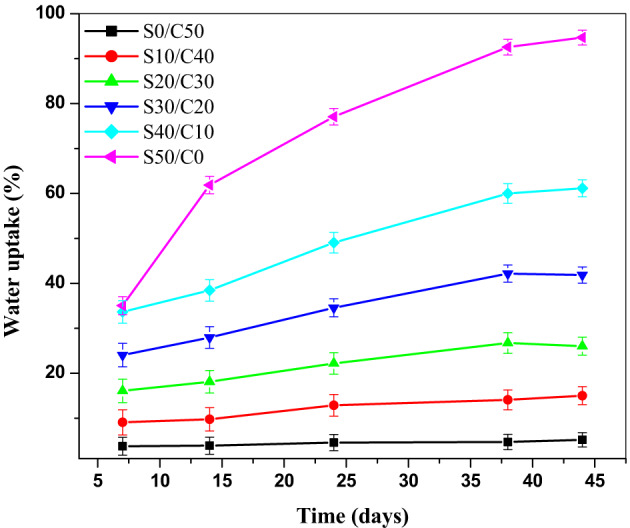
Water uptake of SBR loaded with various concentrations of jojoba seed waste/carbon black hybrid filler.

The water uptake of SBR loaded with various concentrations of jojoba seed waste/carbon black hybrid filler is studied. The obtained results are shown in Fig. 6. It is observed that the water uptake value (%) increases by increasing the percent of seed in the hybrid filler. The value of water uptake is in the order of S50/C0 >S40/C10 >S30/C20 >S20/C30 >S10/C40 >S0/C50. This may be attributed to the hydrophilicity formed by the presence of the hydroxyl groups of jojoba seed waste. This leads to the formation of hydrogen bonds between the seed and water leading to an increase in the absorption of water^31^. It is also observed that the steady state was around 38 days for all the vulcanizates.

### Fourier transforms infrared spectroscopy

**Fig. 7:**
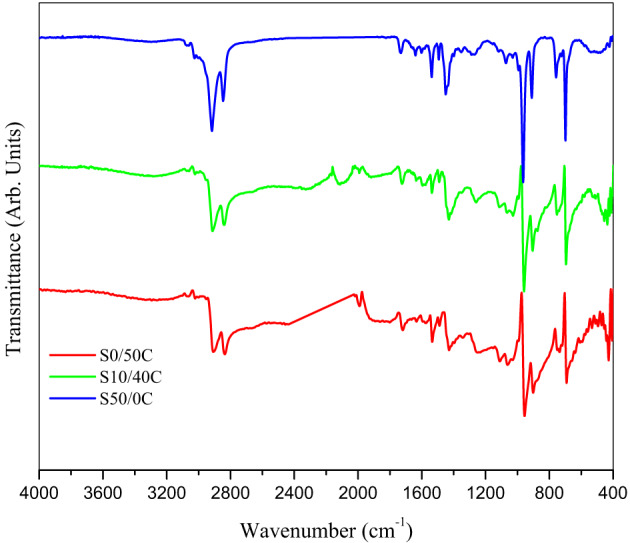
FTIR of SBR composites; S0/50 C; S10/40 C; S50/0 C.

The FTIR spectra of SBR composites containing a blend of jojoba seed waste and carbon black (CB) are shown in Fig. 7. It exhibits distinctive vibrations of the elastomer matrix, including symmetric and asymmetric –C–H stretching (~ 2900 and 2800 cm^−1^), vinyl –C–H out-of-plane bending (~ 980 cm^−1^), and aromatic C–H bending (~ 670 cm^−1^) associated with the polystyrene component^38,39^. The spectra for samples containing CB (such as S0/50 C and S10/40 C) exhibit additional bands in the 2000–1600 cm^−1^ region, indicating that the carbon surfaces have undergone oxidation and possess C = C and C = O functional groups^40–42^. The comparative spectral analysis further substantiated the contributions of each filler. Carbon black exhibited broad signals between 1700 and 3400 cm^−1^, indicating the presence of hydroxyl and carbonyl groups^40–42^. The absorption bands of jojoba seed waste were seen at 1740 cm^−1^ (ester C = O), 2850–2920 cm^−1^ (aliphatic CH_2_), and 1160 cm^−1^ (C–O stretching), consistent with its waxy, lignocellulosic composition^14,43^. As the quantity of seed waste escalated (for instance, S10/40 C compared to S0/50 C), the intensity of the ester and C–O bands intensified, whereas the –OH extending about 3400 cm^–1^ broadened. This indicated that the rubber matrix included enhanced hydrogen bonds and polar interactions. As the loadings increased, the aromatic deformation signals (~ 700–900 cm^−1^) also intensified, indicating that phenolic and lignin-derived components are actively engaging with the polystyrene segments^44^.

**Scheme 1:**
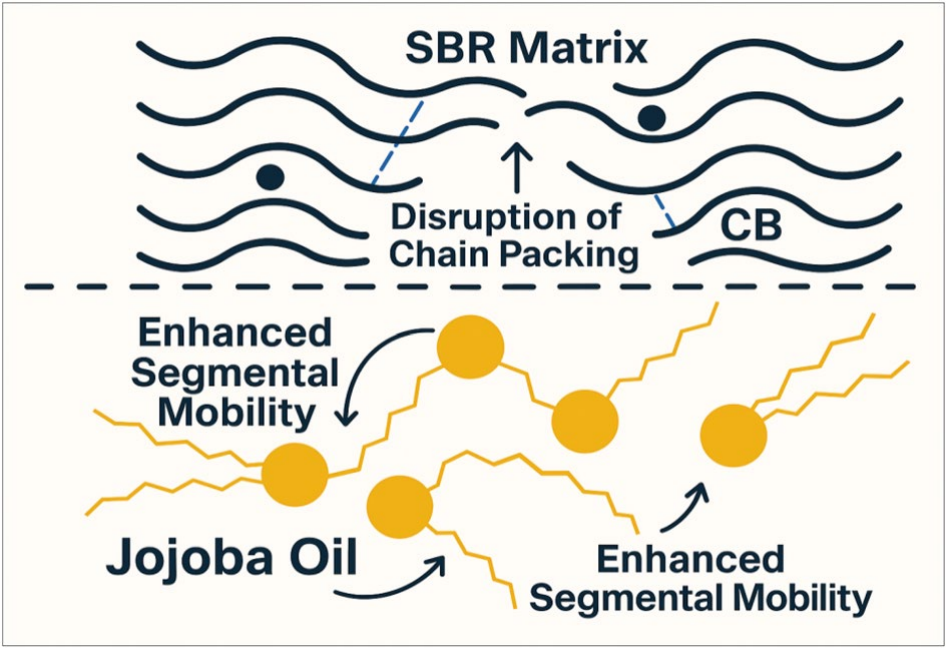
Molecular Schematic of Jojoba Oil Plasticization and Carbon Black Reinforcement in SBR Matrix.

The alterations in the spectra substantiate the notion that two mechanisms govern the behavior of composites. Jojoba oil infiltrates elastomer chains, altering the packing and reducing intermolecular cohesion, as evidenced by variations in the FTIR spectrum. This increases the free volume and segmental mobility. Simultaneously, CB enhances rigidity by adhering to the matrix; however, its reinforcing effect diminishes in the presence of excessive jojoba seed waste due to isolation of the interface. This interaction induces mechanical and dielectric trends that are nonlinear, with plasticization effects intensifying at higher oil loadings. The provided schematic representation (Scheme 1) visualizes these molecular dynamics, illustrating chain disruption by jojoba oil and localized reinforcement zones induced by CB particles^45^.

### Surface morphology through field emission scanning electron microscope

**Fig. 8:**
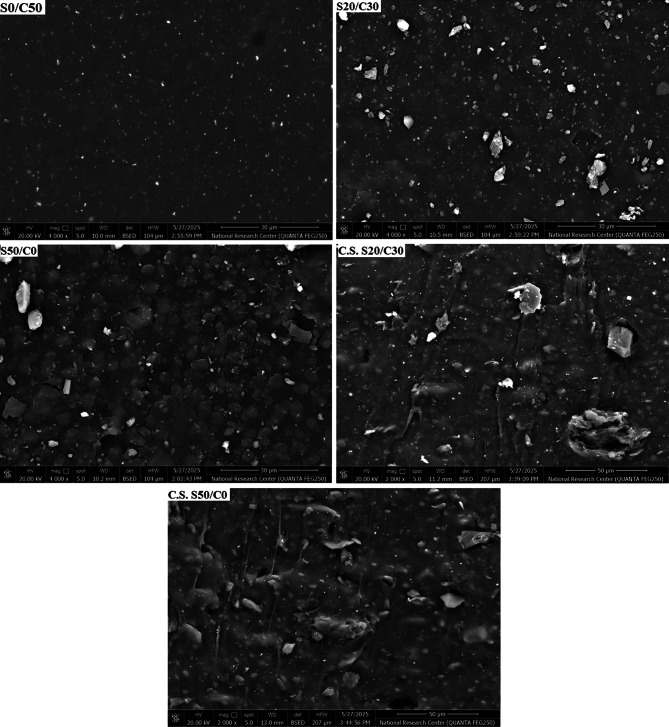
Surface and cross-section images of the SBR composites.

Surface and cross-section images of the prepared SBR composites; namely, S0/C50, S20/C30 and S50/C0 are obtained using FESEM as represented in Fig. 8. As illustrated in the Fig. 8, there are some homogeneous images indicating the good distribution of the components during mixing of the samples. For the sample S0/C50, this sample contains carbon black only. The surface is smooth and has many spots in homogeneous distribution indication for the rubber ingredients. In this sample, carbon black filler is well distributed within the sample, and this explains why this sample has improved mechanical properties. For the sample, S20/C30, it contains 20 phr of jojoba seed and 30 phr of carbon black. The surface has more additional rocks indicating the presence of the jojoba seed and the distribution of the hybrid filler becomes less, and this affects the mechanical properties and leads to a decrease in their improvement rate due to the presence of the seed in the hybrid filler. The image S50/C0 contains jojoba seed only, and no carbon black is present in the sample. By increasing the seed ratio, the distribution of the filler becomes less homogeneous and agglomerations appear and this leads to a further decrease in the mechanical properties. This matches with the results obtained from the mechanical properties. The surface seems to have a sticky nature and this is proved by the images of the cross-section. The cross-section (C.S.) of the sample S20/C30 and the sample S50/C0 reveals the presence of a sticky nature indicating the presence of jojoba seed. This property increases by increasing jojoba seed as observed in the C.S. of S50/C0 sample and this goes hand in hand with the results obtained from elongation that increases by increasing the jojoba seed percentage as a result for the presence of some proportions of jojoba oil.

### Dielectric properties of the vulcanized styrene butadiene rubber composites loaded with Jojoba seed waste powder /carbon black hybrid filler

In order to effectively emphasize the influence of jojoba seed waste/carbon black hybrid filler on the dielectric properties of the SBR composites, it is necessary to investigate the connection between the permittivity (ε′), the dielectric loss (ε″), and the electrical conductivity (σ) at room temperature 30 °C. Comparing SBR composites filled with jojoba seed waste/carbon black hybrid fillers to neat SBR is essential for interpreting the dielectric improvements. The literature indicates that unfilled SBR exhibits low permittivity (ε′ ≈ 2.5–3.2) and low dielectric loss (ε″ ≈ 0.05–0.08) at 100 Hz under standard conditions, with a loss tangent (tan δ) ≤ 0.03^46^. These baseline values indicate that SBR is nonpolar, serves as an insulator, and possesses a low density of mobile charges. Conversely, using hybrid fillers, particularly conductive carbon black and polar functionalities from lignocellulosic seed components, significantly increases ε′ and ε″.

**Fig. 9:**
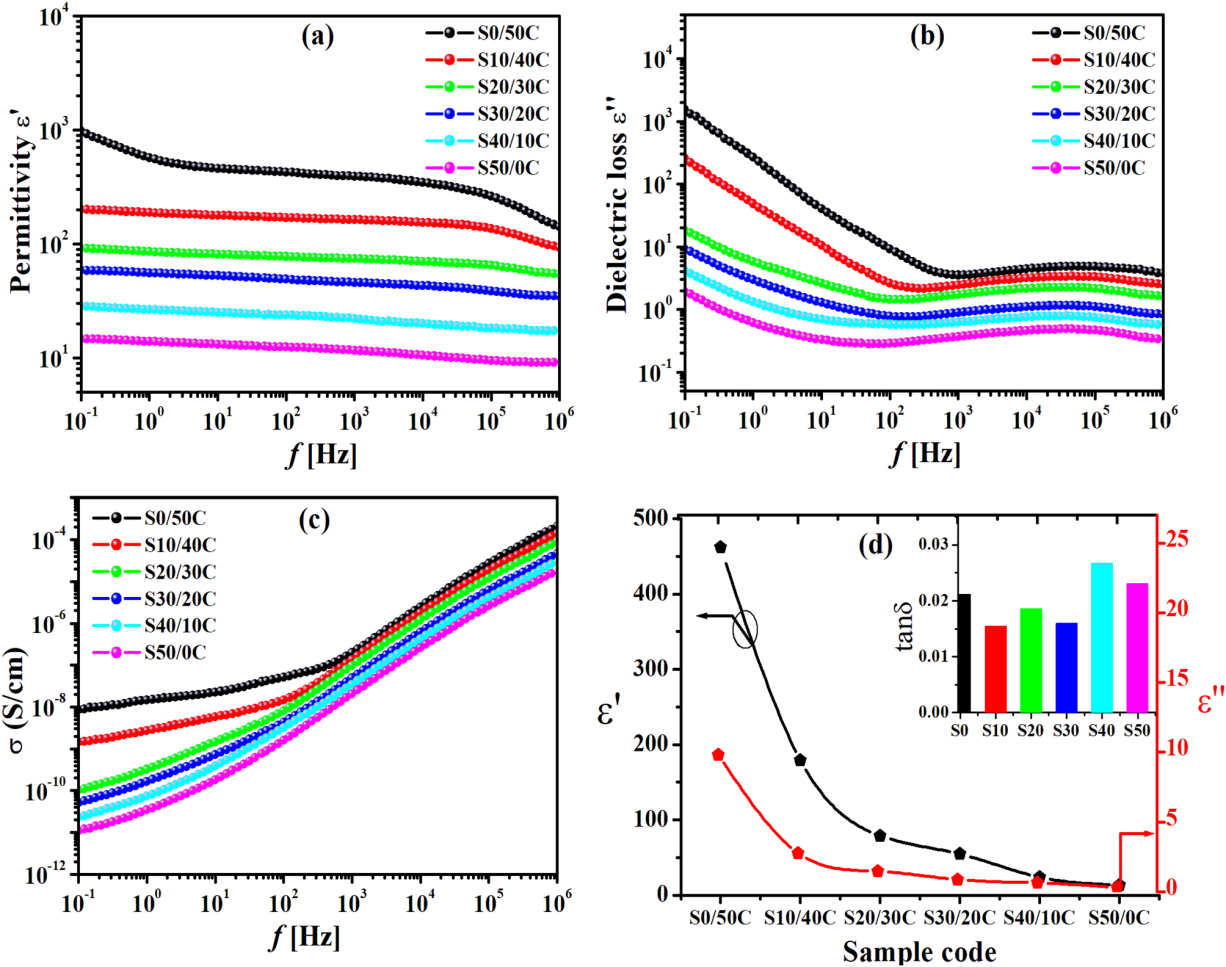
Frequency dependence of; (a) permittivity (ε′), (b) the dielectric loss (ε″), (c) the electrical conductivity (σ) for SBR loaded with various concentrations of jojoba powder/carbon black hybrid filler. (d) Changes in permittivity (ε′), dielectric loss (ε″) and loss tangent tanδ (the insert) as a function of hybrid filler loading at 100 Hz.

Figure 9a illustrates that the permittivity (ε′) of SBR loaded with jojoba seed powder /carbon black hybrid filler exhibits a decrease with increasing seed content. A slight frequency dependency is noticed when the concentration of seed in the hybrid filler is below 20 phr till reaching 50 phr. The permittivity (ε′) of the composites decreases with the rising number of seed across the entire frequency spectrum. For example, the permittivity (ε′) of SBR/carbon black (S0/50 C) composite at 100 Hz is approximately 462, which is reduced to 13.4 for SBR/seed (S50/0 C) composite (Fig. 9d). The significant decrease in ε′ can be ascribed to the reduced ability of the composite to store electrical energy arising from aggregation of fillers. Increased seed loadings may result in agglomeration, diminishing the effective surface area for polarization and hence decreasing the permittivity^47^. However, interfacial polarization significantly affects the dielectric characteristics; interfacial polarization, namely Maxwell-Wagner-Sillars (MWS) polarization^48,49^, arises from the accumulation of charges at the phase interfaces among the hybrid filler, and the polymer matrix. Specifically, for lower seed loadings, interfacial polarization raises the permittivity (ε′), whereas as seed loading grows, the impact of interfacial polarization diminishes, resulting in a drop in ε′. For carbon black-dominant systems (50/0 and 40/10), the hybrid filler compositions (50/0 and 40/10) exhibit elevated values of ε′ and ε″, especially at lower frequencies, attributed to significant MWS interfacial polarization. The conductive properties of carbon black and its fine dispersion facilitate charge accumulation at the filler–matrix interfaces, resulting in significant field-induced polarization and improved dielectric activity. At intermediate hybrid ratios of 30/20 and 20/30 As JSMP content increases, both ε′ and ε″ exhibit a gradual decrease. JSMP interferes with CB conductive pathways and diminishes the dielectric contrast required for significant interfacial polarization. The dielectric response exhibits increased frequency dependence, while polarization effects diminish, signifying a shift towards insulating behavior. On the other hand, JSMP-dominant systems (10/40 and 0/50). The samples exhibit the minimum ε′ and ε″ values throughout the frequency spectrum. The bio-based filler demonstrates low conductivity and incorporates natural waxes that diminish charge retention at interfaces. Agglomeration at elevated loadings may contribute to morphological saturation, reducing the available interfacial area and inhibiting MWS polarization^50–53^.

The dielectric loss (ε″), of the hybrid composites exhibits frequency dependency, as illustrated in Fig. 9b. The value of ε″ of all samples exhibits a significant rise at lower frequencies. However, it increased immediately beyond 10^3^ Hz, which can be attributed to dipole orientation. The dipole lacked sufficient time for orientation^54^. Generally, SBR composites exhibit a notable decrease in the dielectric loss (ε″) with an increase in seed content in the hybrid filler (Fig. 9d), attributable to the synergistic effects of the hybrid fillers^55^.

**Fig. 10:**
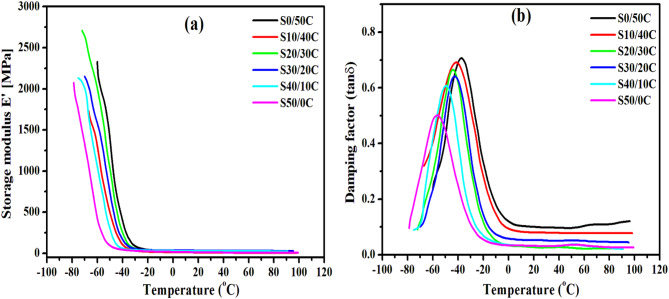
The Storage modulus (E′), and the damping factor (tanδ) versus temperature for SBR loaded with different concentrations of jojoba seed/carbon black hybrid filler.

On the other hand, the conductivity of all samples diminishes with an increase in seed content and rises with an increase in frequency following power law^56^, as depicted in Fig. 9c. The conductivity of the SBR/carbon black (S0/50 C) composite is around ~ 10^− 8^ S/cm, which decreases to ~ 10^− 11^ S/cm for the (S50/0 C) composite. This indicates that the conductivity of the hybrid composites falls within the specified range (10^− 8^- 10^− 11^ S/cm). The significant reduction in conductivity (σ) with increased seed concentration indicates an improvement in the insulating characteristics of the SBR composites. Curiously, when the seed concentration increases, the composites permittivity (ε′) drops from 462 to 13.4 in Fig. 9d. Alternatively, the loss tangent (tanδ), as shown in the Fig. 10b inset, stays below 0.03 regardless of the filler concentration, suggesting that there is minimal energy dissipation. It can be concluded that the hybrid filler has the ability to impart a high permittivity and a low loss tangent (tanδ) to the SBR.

### Dynamic mechanical characteristics of styrene butadiene rubber composites containing Jojoba seed waste/carbon black hybrid filler

The incorporation of hybrid fillers into styrene-butadiene rubber alters its dynamic properties. This becomes evident when compared with pristine sample. The unfilled sample often demonstrates a storage modulus E of 7–12 MPa at 25 °C, a glass transition temperature (Tg) ranging from − 45 °C to − 50 °C, and a maximum damping factor tan δ_max_ below 0.65, indicating moderate stiffness and unrestricted segmental motion^46^. These baseline values serve as a reference for assessing alterations induced by fillers. However, for a better understanding of the viscoelastic properties of these composites, it is vital to link both the temperature and the concentration of the hybrid filler. Figure 10 illustrates the storage modulus (E′) and damping factor (tan δ) of styrene butadiene rubber hybrid composites as functions of temperature (-80 to 100 °C). The results of both criteria are summarized in Table 3. E′ displays three temperature-dependent regions: a low-temperature glassy zone, a narrow sharp decline corresponding to relaxation in the polymer matrix, and a high-temperature rubbery plateau.

Upon increasing the temperature, E′ (Fig. 10a) exhibits a significant decline between − 60 and − 30 ◦C, signifying a change from a glassy to a rubbery state. The micro-Brownian motion of polymeric chains around Tg reduces the storage modulus value^57^. Consequently, as the temperature rises, molecule mobility escalates; thus, the densely packed structure relinquishes its compact arrangement, resulting in a reduction of E′. Nonetheless, no noteworthy changes in trends were detected for the hybrid filler-loaded composites in comparison to the carbon- loaded one. It is found that, E′ reduced upon rising seed content in the hybrid filler. This results from the excessive buildup of fillers, leading to the production of agglomerates. The existence of filler clusters generates substantial voids inside the SBR matrix, significantly enhancing polymer segmental and rotational mobility and reducing E′.

The damping factor (tan δ) represents the ratio of E′ to E″ and typically characterizes the elastic and viscous properties of a polymer system^58^. The peak height indicates the internal energy dissipation at the filler/polymer interphase. In Fig. 10b represents the tan δ curves for SBR composites as a function of temperature. The tan δ curves indicate that with increasing temperature, the damping factor rises in the glassy region, attains a maximum in the transition region, and subsequently declines in the rubbery region. Increasing seed loadings reduced tan δ maximum as it lowers the friction at the contact between the filler and the matrix, as the seed are soft and derived from plants. Consequently, this reduces energy dissipation and, thus, damping intensity^59,60^, indicating a reduced level of molecular mobility. Additionally, it shifts the tan δ peak to a lower temperature, which typically denoted by a reduction in the glass transition temperature Tg of the SBR composites. The naturally occurring waxes and oils in seed serve as internal plasticizers when integrated into the matrix. This procedure improves the mobility of the segmental chain, promoting relaxation processes and lowering the damping peak temperature. Moreover, Jojoba seed may disrupt sulfur-based crosslinking chemistry, particularly in high quantities, hence reducing the density of the crosslinks as discussed earlier. The obtained results align with prior studies^61,62^.

Table 4 demonstrates that the storage modulus E′ at 25 °C and the glass transition temperature Tg, decreases with the rising of seed concentration of SBR hybrid composites. The reduction in storage modulus with higher hybrid filler concentration is a prevalent phenomenon in composite materials. The incorporation of hybrid fillers into a polymer matrix can interfere with the polymer chains, resulting in diminished material stiffness. This leads to a diminished storage modulus, which quantifies the material’s capacity to retain elastic energy^63^. The glass transition temperature Tg, decreases with increasing seed content may result from plasticization and decreased crosslinking, which may directly affect the mobility of the polymer chains.

**Table 4:** The storage modulus (E’), glass transition temperature (Tg) and maximum loss factor (tan δ_max_) of SBR composites.

Sample	(E’) @ 25 °C	Tg (°C)	tanδ_max_
S0/50C	18.8	-37.4	0.71
S10/40C	17.0	-41.1	0.69
S20/30C	17.9	-44.8	0.68
S30/20C	15.3	-42.3	0.63
S40/10C	12.6	-49.7	0.64
S50/0C	11.3	-55.5	0.52

**Fig. 11:**
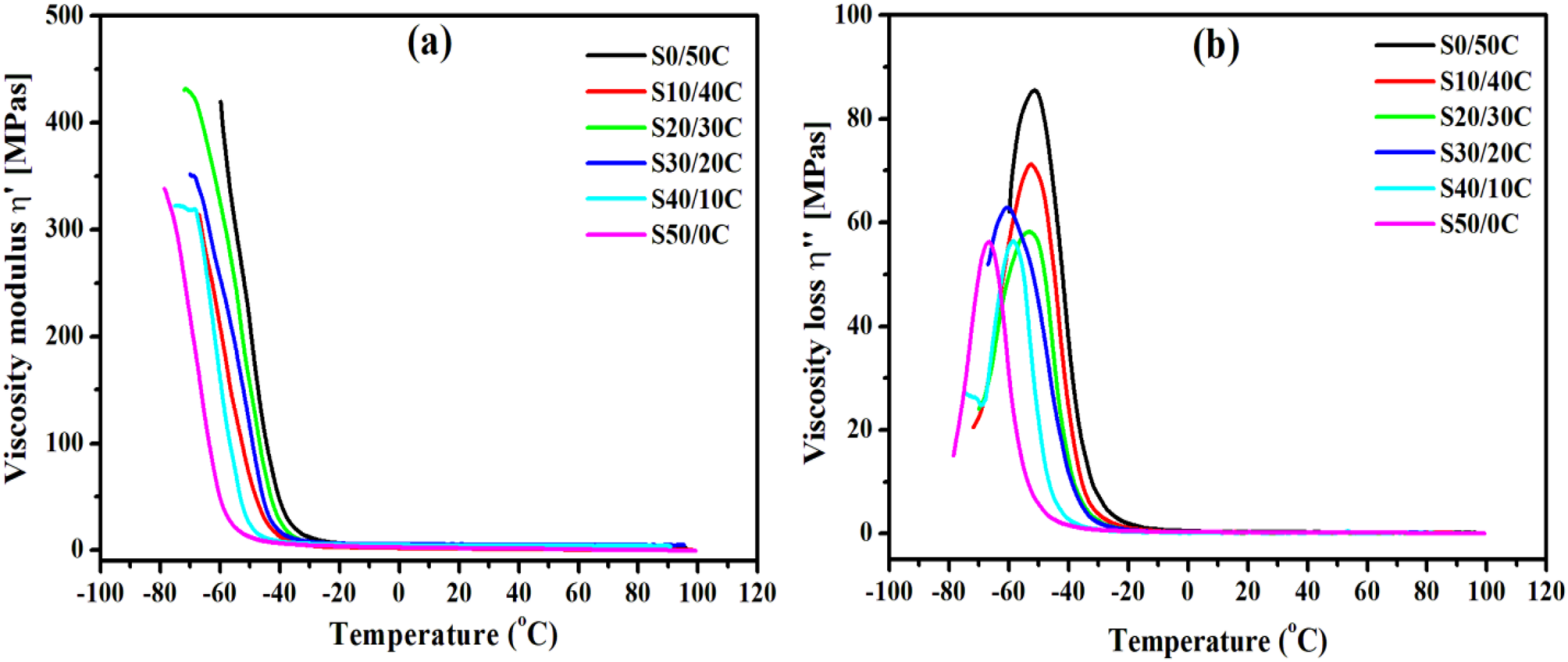
The real and imaginary components of complex viscosity versus temperature for SBR composites loaded with different concentrations of jojoba seed/carbon black hybrid filler.

Figures 11(a) and (b) depict the real and imaginary components of complex viscosity, respectively. Figure 11(a) illustrates the correlation between the flow characteristics of the rubber matrix and temperature. The inclusion of seed powder in the SBR matrix leads to a decrease in the real and imaginary components of complex viscosity. The plasticized effect of jojoba oil^33^ may be responsible for this trend. At low temperatures, the SBR composites display increased rigidity and a glassy state, as indicated by elevated viscosity modulus values resulting from restricted molecular mobility. As temperature rises and nears the glass transition temperature (Tg), the material shifts to a rubbery state, characterized by increased mobility of molecular chains. The transition is characterized by a significant reduction in the viscosity modulus, and loss modulus indicating increased flexibility and decreased stiffness of the composite at elevated temperatures. An important factor that leads to the large reduction in viscosity is the improved chain mobility that is brought by growing seed powder concentration that contains a high concentration of jojoba oil. On the other hand, the obtained results are consistent with the other physical properties that are investigated earlier. In addition, these materials are advantageous for applications that require materials to maintain their flexibility and performance throughout a broad temperature range.

## Conclusion

This study investigates the impact of jojoba seed waste powder/carbon black as hybrid filler on the mechanical, dielectric, and viscoelastic properties of the SBR composites. Jojoba powder reduces the minimum viscosity (M_L_) and scorch time (TS_2_), while enhancing crosslinking up to a maximum of 40 phr. Excessive Jojoba powder (50 phr) results in agglomeration and extended curing duration. The carbon black composite possesses the maximum tensile strength independently; however, including more seed renders the combination more plastic, thereby lessening its strength and hardness while enhancing its flexibility. The aging process deteriorates tensile properties while enhancing modulus due to the occurrence of crosslinking over time. As the quantity of seed rises, the swelling behavior intensifies while the crosslink density reduces. This behavior demonstrates equilibrium between adaptability and structural stability. On the other hand, the dielectric investigation indicates that elevated seed concentration disrupts polarization, resulting in reduced permittivity (ε′) and conductivity (σ), hence enhancing insulation. Increased dielectric loss (ε″) at low frequencies indicates enhanced energy dissipation. The dynamic mechanical analysis reveals that seed-rich composites demonstrate a decreased storage modulus (E′) and glass transition temperature (Tg). Jojoba powder generates voids, facilitating increased molecular mobility. Furthermore, both the real and imaginary components of complex viscosity (η*) decrease, indicating that the material is suitable for flexible applications throughout a broad temperature spectrum. The versatile properties of these hybrid composites render them suitable for tire treads, gaskets, industrial seals, and cable insulation. Their ability to adapt, resist aging, and provide superior insulation compared to other materials indicates their potential utility across various technical domains.

## Supplementary Information

Below is the link to the electronic supplementary material.

Supplementary Material 1

## Data Availability

The datasets used and/or analysed during the current study available from the corresponding author on reasonable request.
