# Supplementary material for: Influence of Jojoba seed waste and carbon black hybrid filler on styrene butadiene rubber composites characteristics
Source: Sci Rep. 2025 Oct 17;15:36302. doi: 10.1038/s41598-025-21649-4 (PMC12534392; doi:10.1038/s41598-025-21649-4)
Supplement: Supplementary file 1 — Supplementary Material 1 [file 41598_2025_21649_MOESM1_ESM.docx]

**Descriptive Statistical Analysis of Composite Performance**

To ensure objectivity in the interpretation of experimental trends represented in Figures 1 to 5 in the manuscript, descriptive statistical metrics—including mean values, standard deviations, and observed ranges—were calculated for all key mechanical and physico-chemical properties. These metrics help quantify the extent of variability and establish consistency within each formulation set. Some statistics are listed in Supplementary Tables (S1-S9) as follows:

**Mechanical Properties**

- **Tensile Strength** recorded a mean of 10.2 MPa, with a considerable range from 1.42 MPa to 18.0 MPa (SD: 6.86 MPa), pointing to heterogeneous load-bearing capabilities likely driven by differences in filler dispersion and interfacial bonding.
- **Elongation at Break** showed high ductility (mean: 1.06 ×10^3^ %; SD: 313 %), although the minimum value of 668 % suggests localized network restriction or filler agglomeration in certain samples.
- **Modulus** ranged from 0.56 to 2 MPa, averaging at 1.09 MPa (SD: 0.58 MPa), reflecting the composite stiffness and its dependence on crosslink density.
- **Hardness** was comparatively stable, with a mean of 70.0 Shore A and SD of 9.61, highlighting moderate filler-induced influence on surface characteristics.

Supplementary Table S1: Statistics of tensile strength

| Statistics of tensile strength | Value |
| --- | --- |
| Mean (Average) | 10.2 |
| Standard deviation | 6.86 |
| Minimum | 1.42 |
| Maximum | 18.1 |

Supplementary Table S2: Statistics of elongation

| Statistics of elongation | Value |
| --- | --- |
| Mean (Average) ×10^3^ | 1.06 |
| Standard deviation | 313 |
| Minimum | 668 |
| Maximum×10^3^ | 1.40 |

Supplementary Table S3: Statistics of modulus

| Statistics of modulus | Value |
| --- | --- |
| Mean (Average) | 1.09 |
| Standard deviation | 0.58 |
| Minimum | 0.56 |
| Maximum | 2 |

Supplementary Table S4: Statistics of hardness

| Statistics of hardness | Value |
| --- | --- |
| Mean (Average) | 70.0 |
| Standard deviation | 9.61 |
| Minimum | 58.7 |
| Maximum | 84.5 |

#### **Ageing Behavior**

Time-dependent changes in mechanical integrity were tracked across four ageing durations (room temperature, 2, 4, and 6 days):

- **Tensile Strength** declined from 10.2 MPa (RT) to 6.45 MPa (6 days), while SD reduced from 6.85 MPa to 3.60 MPa, suggesting progressive material homogenization or degradation.
- **Elongation at Break** decreased markedly from 1.06 ×10^3^ % to 453%, reflecting embrittlement, potentially due to oxidative chain scission or enhanced crosslinking.
- **Modulus Post-Ageing** increased from 1.09 MPa to 2 MPa, indicating stiffening. However, the large SD of 2.43 MPa at 6 days suggests oxidative processes may have produced non-uniform effects.

Supplementary Table S5: Statistics of tensile after ageing

| Statistics of tensile after ageing | Value |
| --- | --- |
| Mean (Average) | 10.2 (r.t.), 9.56 (2 days), 7.52 (4 days), 6.45 (4 days) |
| Standard deviation | 6.85 (r.t.), 6.08 (2 days), 4.32 (4 days), 3.60 (6 days) |
| Minimum | 1.42 (r.t.), 2.02 (2 days), 1.75 (4 days), 1.86 (6 days) |
| Maximum | 18.0 (r.t.), 16.5 (2 days), 12.3 (4 days), 10.1 (6 days) |

Supplementary Table S6: Statistics of elongation after ageing

| Statistics of elongation after ageing | Value |
| --- | --- |
| Mean (Average) ×10^3^ | 1.06 (r.t.), 1.03 (2 days), 0.72 (4 days), 0.45 (6 days) |
| Standard deviation | 313 (r.t.), 408 (2 days), 526 (4 days), 242(6 days) |
| Minimum | 698 (r.t.), 601 (2 days), 261 (4 days), 164 (6 days) |
| Maximum ×10^3^ | 1.40 (r.t.), 1.54 (2 days), 1.57 (4 days), 0.81 (6 days) |

| Statistics of modulus after ageing | Value |
| --- | --- |
| Mean (Average) | 1.09 (r.t.), 1.67 (2 days), 2.03 (4 days), 2 (6 days) |
| Standard deviation | 0.54 (r.t.), 0.48 (2 days), 0.66 (4 days), 2.43(6 days) |
| Minimum | 0.56(r.t.), 1.21 (2 days), 1.33 (4 days), 2.97 (6 days) |
| Maximum | 1.40 ×10^3^ (r.t.), 1.54 ×10^3^ (2 days), 1.52 (4 days), 3.59 (6 days) |

Supplementary Table S7: Statistics of modulus after ageing

#### **Network Integrity and Swelling Behavior**

- **Equilibrium Swelling** values (mean: 284; SD: 74.8) suggest varied solvent uptake capacity, indirectly tied to network permeability.
- **Crosslink Density**, estimated from swelling data, averaged 1.33 × 10⁻⁴ mol/cm³ with a high SD of 8.51 × 10⁻⁵, signifying notable compositional and microstructural diversity among formulations.

Supplementary Table S8: Statistics of equilibrium swelling

| Statistics of equilibrium swelling | Value |
| --- | --- |
| Mean (Average) | 284 |
| Standard deviation | 75 |
| Minimum | 172 |
| Maximum | 352 |

Supplementary Table S9: Statistics of crosslink density

| Statistics of crosslink density | Value |
| --- | --- |
| Mean (Average) | 1.33 × 10^-4^ |
| Standard deviation | 8.51 × 10^-5^ |
| Minimum | 7.03 × 10^-5^ |
| Maximum | 2.84 × 10^-4^ |
